# High-Performance Conducting Polymer Nanotube-based Liquid-Ion Gated Field-Effect Transistor Aptasensor for Dopamine Exocytosis

**DOI:** 10.1038/s41598-020-60715-x

**Published:** 2020-02-28

**Authors:** Seon Joo Park, Jiyeon Lee, Sung Eun Seo, Kyung Ho Kim, Chul Soon Park, Sang Hun Lee, Hyun Seung Ban, Byoung Dae Lee, Hyun Seok Song, Jinyeong Kim, Chang-Soo Lee, Joonwon Bae, Oh Seok Kwon

**Affiliations:** 1 Infectious Research Center, Korea Research Institute of Bioscience & Biotechnology (KRIBB), Daejeon, 34141 Republic of Korea; 2 Department of Neuroscience, Graduate School, Kyung Hee University, Seoul, 02447 Republic of Korea; 3 Department of Bioengineering, University of California Berkeley, Berkeley, 94720 CA USA; 4 Biotherapeutics Translational Research Center, Korea Research Institute of Bioscience & Biotechnology (KRIBB), Daejeon, 34141 Republic of Korea; 5 Department of Physiology, Kyung Hee University School of Medicine, Seoul, 02447 Republic of Korea; 6 Sensor System Research Center, Korea Institute of Science and Technology, Seoul, 02792 Republic of Korea; 7 Bionanotechnology Research Center, Korea Research Institute of Bioscience & Biotechnology (KRIBB), Daejeon, 34141 Republic of Korea; 8 Biotechnology (Nanobiotechnology), University of Science & Technology (UST), Daejeon, 34141 Republic of Korea; 9 Department of Applied Chemistry, Dongduk Women’s University, Seoul, 02748 Republic of Korea

**Keywords:** Chemistry, Polymer synthesis

## Abstract

In this study, ultrasensitive and precise detection of a representative brain hormone, dopamine (DA), was demonstrated using functional conducting polymer nanotubes modified with aptamers. A high-performance aptasensor was composed of interdigitated microelectrodes (IMEs), carboxylated polypyrrole nanotubes (CPNTs) and DA-specific aptamers. The biosensors were constructed by sequential conjugation of CPNTs and aptamer molecules on the IMEs, and the substrate was integrated into a liquid-ion gating system surrounded by pH 7.4 buffer as an electrolyte. To confirm DA exocytosis based on aptasensors, DA sensitivity and selectivity were monitored using liquid-ion gated field-effect transistors (FETs). The minimum detection level (MDL; 100 pM) of the aptasensors was determined, and their MDL was optimized by controlling the diameter of the CPNTs owing to their different capacities for aptamer introduction. The MDL of CPNT aptasensors is sufficient for discriminating between healthy and unhealthy individuals because the total DA concentration in the blood of normal person is generally determined to be *ca*. 0.5 to 6.2 ng/mL (3.9 to 40.5 nM) by high-performance liquid chromatography (HPLC) (this information was obtained from a guidebook “Evidence-Based Medicine 2018 SCL “ which was published by Seoul Clinical Laboratory). The CPNTs with the smaller diameters (CPNT2: *ca*. 120 nm) showed 100 times higher sensitivity and selectivity than the wider CPNTs (CPNT1: *ca*. 200 nm). Moreover, the aptasensors based on CPNTs had excellent DA discrimination in the presence of various neurotransmitters. Based on the excellent sensing properties of these aptasensors, the DA levels of exogeneous DA samples that were prepared from PC12 cells by a DA release assay were successfully measured by DA kits, and the aptasensor sensing properties were compared to those of standard DA reagents. Finally, the real-time response values to the various exogeneous DA release levels were similar to those of a standard DA aptasensor. Therefore, CPNT-based aptasensors provide efficient and rapid DA screening for neuron-mediated genetic diseases such as Parkinson’s disease.

## Introduction

DA is an important mammalian brain hormone and chemical neurotransmitter that regulates the functions of the central nervous and cardiovascular systems. The major reason for fluctuations in DA levels is a loss of dopaminergic neurons in the substantia nigra pars compacta. A fluctuation in DA concentration in living bodies might result in several formidable diseases, such as Parkinson’s and Alzheimer’s diseases^1–6^. Therefore, it is extremely important to detect and monitor DA levels in human bodies for clinical diagnosis and disease prevention.

Aptamers are artificial nucleic acid such as DNA or RNA ligands synthesized by systematic evolution of ligands by exponential enrichment (SELEX) process^7,8^. They have provided high-affinity and specificity against a wide variety of receptors (probes or capture molecules), including small organics, peptides, proteins and complex molecules. In addition, aptamers can recognize non-immunogenic and toxic targets. Therefore, one of the next generation biosensors is an aptasensor which employ aptamers as a recognition element. There are many kinds of aptasensors such as electrochemical aptasensors, fluorescence aptasensors and FETs, which have different sensing mechanisms. Interestingly, the immobilized aptamers on the electrical transistor enable to make conformation changes with target molecules, resulted into the electrical signal detection. In addition, the aptasensors provide high-affinity and superior stability compared to conventional receptors such as peptides, proteins and antibodies^8^. Therefore, the aptasensors are used for high-performance biosensors which are also required for ultra-high environmental stability with resistance to degradation and denaturation.

Conducting polymer nanomaterials with various advantages, such as easy preparation, cost effectiveness, and biocompatibility, have been highlighted in many applications, such as transparent electrodes, antistatic coatings, electromagnetic shielding materials, light emitting devices, batteries, photovoltaics, and sensors^9–15^. For example, nanoscale materials prepared from polypyrrole (PPY), polyaniline, and polythiophene and their derivatives have been extensively used for diverse purposes^16–21^. In addition, conducting polymer nanomaterials are also very attractive materials for sensing purposes because they can effectively deliver electrical sensing signals due to facilitated charge transport along molecular chains owing to conjugated double bonds^22^. In particular, the chemical and dimensional stability of PPY nanomaterials is desirable for the introduction of external moieties, such as binding groups or aptamers, by surface modification. More specifically, the use of PPY and its derivatives for sensing purposes has been conceptually popular.

To date, numerous strategies and materials have been introduced to generate ultrasensitive DA sensors using PPY and other components, such as nanocrystals, carbon nanostructures, and molecular imprinted polymers. For example, diverse PPY nanostructures, such as hexagonal-shaped particles, could be applied as sensing media^23,24^. The addition of nanocrystals was advantageous due to the combination of the unique properties from both PPY and nanocrystals^25–29^. Carbon nanostructures showing good mechanical robustness and electrical conductivity, especially carbon nanotubes, could be widely used for DA screening. However, DA showed high nonspecific binding effects with significant signal-to-noise levels because the benzene-ring structures of DA can easily adsorb on the surface of carbon nanotubes. Therefore, carbon nanotubes were incorporated into a PPY matrix to develop DA specificity and sensitivity^30–35^. In some cases, PPY/carbon/nanocrystal three-component sensing materials exhibited increased sensitivity in sensors^36^. There are various strategies using only conducting polymer nanomaterials for DA detection. Molecularly imprinted PPY was also employed in electrochemical sensors^37^. PPY and other components were introduced on the surfaces of various electrodes *via* interfacial engineering techniques^38,39^. Interestingly, the nanohybrid material carboxylated PPY-coated carbon nanotubes were designed and showed DA agonism and antagonism. Considering the previous research activities, it can be inferred that functionalized PPY nanomaterials are very promising as sensing media for DA detection.

On the other hand, variation in sensor geometry is one of the most important parameters for signal detection and sensor performance. Therefore, diverse approaches for DA detection involving sensor geometry based on PPY have been reported. The most representative methods are electrochemical sensing techniques, which are feasible due to the electroactivity of DA molecules^40,41^. FET-type DA sensors have been suggested; however, these sensors have suffered from a relatively slow response and low selectivity because the sensors were not exclusively specific for DA molecules. A critical issue associated with most sensors is low sensitivity, which should be addressed by the introduction of functional groups/molecules that can interact exclusively with DA molecules. A challenging strategy to overcome this issue is the use of aptamers, molecules that are a series of nucleic acids. While the incorporation of aptamers into DA sensors has been reported^42,43^ the introduction of aptamers to PPY nanomaterials has been scarcely reported.

In this study, we first demonstrated a liquid-ion gated FET platform based on aptamer-conjugated CPNTs for exogeneous DA detection. Moreover, to optimize their sensing performance, CPNTs with different diameters were prepared by reverse microemulsion, and the DA aptamer as a gating modulator was chemically attached to the surface of CPNTs. The FET aptasensor showed highly sensitive and selective real-time responses to various concentrations of DA, as a standard reagent: the small-diameter CPNTs reached especially higher sensitivity (more than 100 times greater; 100 pM) than the large-diameter CPNTs. The MDL of CPNT aptasensors was sufficient for discriminating between healthy individuals and patients with DA-related illnesses because high-performance liquid chromatography (HPLC) shows that the blood of the latter group has *ca*. 0.6 to 6.2 ng/mL (3.9 to 40.5 nM) DA (this information was obtained from a guidebook “Evidence-Based Medicine 2018 SCL” which was published by Seoul Clinical Laboratory). Moreover, the aptasensor had excellent specificity in the presence of various neurotransmitters. Based on these excellent sensing performances with FET aptasensors, exogeneous DA samples were successfully prepared from PC12 cells by DA release assay and measured by CPNT aptasensors. DA exocytosis from aptasensors was also identified, and their sensing properties are similar to those of standard DA reagents.

## Results and Discussion

### CPNT-based aptasensor for DA exocytosis

**Figure 1:**
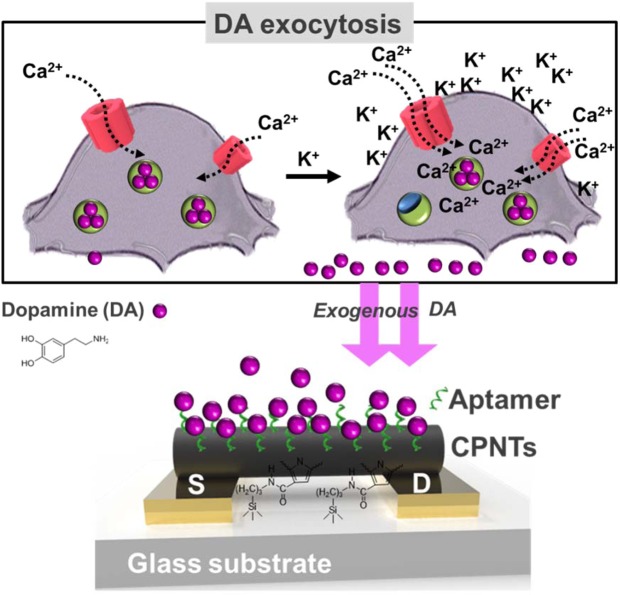
Schematic illustration of cell exocytosis for DA release from PC12 cells via rapid Ca^2+^ reflux accelerated by K^+^ ions (upper) and liquid-ion gated FET aptasensors using aptamer-conjugated CPNTs for exogeneous DA detection (down).

**Figure 2:**
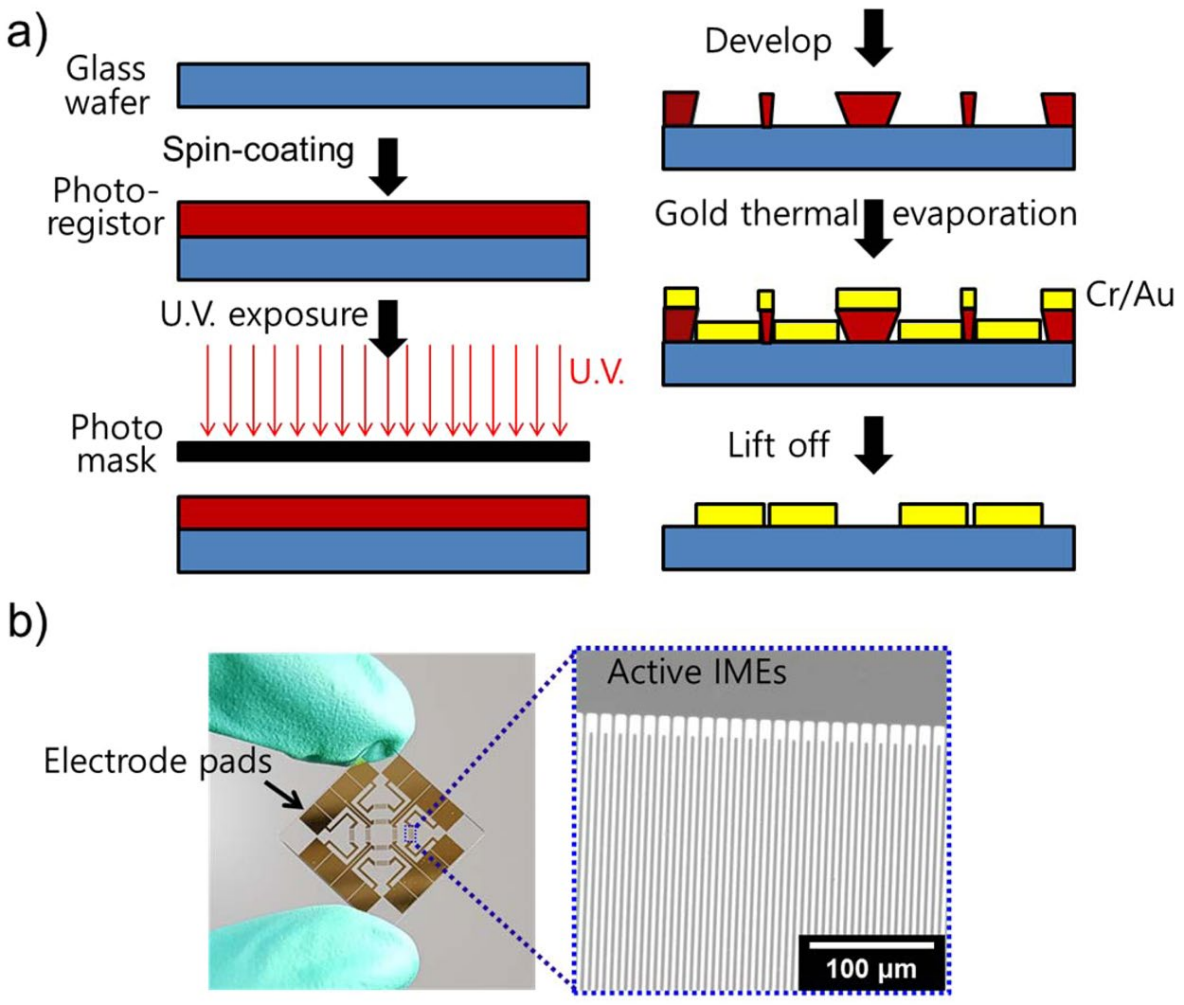
(**a**) Schematic of the processes for IME fabrication. (**b**) Photograph of IMEs and SEM image of the extended active IME array. The prepared IME array was 2 μm wide, 1.5 mm long and 30 nm thick (Au 25/Cr 5 nm).

We designed an electrical methodology for real-time exogeneous DA release detection. Figure 1 shows a representative illustration of the cell-related DA exocytosis process and an FET aptasensor for exogeneous DA monitoring. The DA in cells was released by Ca^2+^ transport through the calcium ion channel, and the Ca^2+^ influx was controlled by the KCl concentration *via* DA release assay. The advantage of KCl addition is that the rate of DA release is rapid compared to the rate in the presence of only Ca^2+^ owing to the enhanced Ca^2+^ influx effect^44^. The exogeneous DA samples were prepared and monitored by liquid-ion gated FET aptasensors consisting of an IME array, CPNTs and aptamers. First, the IME array was prepared by a microelectromechanical system (MEMS) (Fig. 2a). Specifically, the photoresistor was spin-coated onto a glass wafer and exposed to UV light using a patterned photomask. Subsequently, a pattern was developed by the developer, and Au was deposited on the substrate with Cr as a binder by thermal evaporation. A final pattern was obtained after a simple lift-off. A photograph and scanning electron microscopy (SEM) images of the IME array are also presented in Fig. 2b. The Au lines bridged electrode pads, and active IMEs were passivated with a photoresistor to protect against nonspecific binding with nontarget biomolecules and liquid-surrounding contaminants.

**Figure 3:**
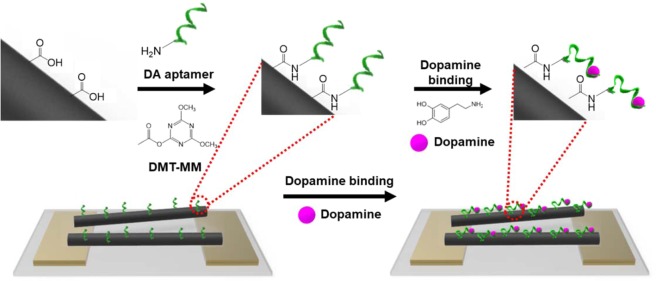
Fabrication protocol of aptasensor based on aptamer-conjugated CPNTs. Chemical conjugation mechanism between CPNTs and aptamer by the condensation reagent DMT-MM.

Generally, the nanomaterials functioning as transistors in biosensors were adsorbed on the IME array because the drop-casting and spin-coating processes are simple and easy. However, the adsorption of nanomaterials on the IME array is easily affected by the surrounding environment, such as liquid fluctuations, and the nanomaterials are detached on the IME array during cleaning or washing processes for introducing aptamers, leading to low reproducibility and sensitivity. Compared to conventional adsorption processes of nanomaterials, in this study, the CPNTs were immobilized on the IME array, which was functionalized with the -NH group by silane coupling agents^45^. The overall construction protocol of the aptasensor is illustrated in Fig. 3. CPNTs were chemically immobilized on the silane-treated IME arrays by a condensation reaction with DMT-MM, resulting in the stable electrical contact between CPNTs and IMEs. The extended illustration in Fig. 3 shows the ideal aptasensor consisting of immobilized CPNT bridges between the source and drain electrodes. The aptamer, which was benchmarked by a previous study, was chemically attached on the surface of CPNTs by the same reaction^46,47^. This benchmarked aptamer consists of DNA sequences, not RNA^48–50^. DNA aptamers have attractive advantages compared to RNA aptamers: 1) they are more stable under various environmental conditions, and 2) they have improved affinity owing to superior DA binding sites. Additionally, DMT-MM, which is representative of chemical reagents used to chemically bind the carboxylic group of nanomaterials and the amine group of bioprobes, was used as a conjugation reagent.

**Figure 4:**
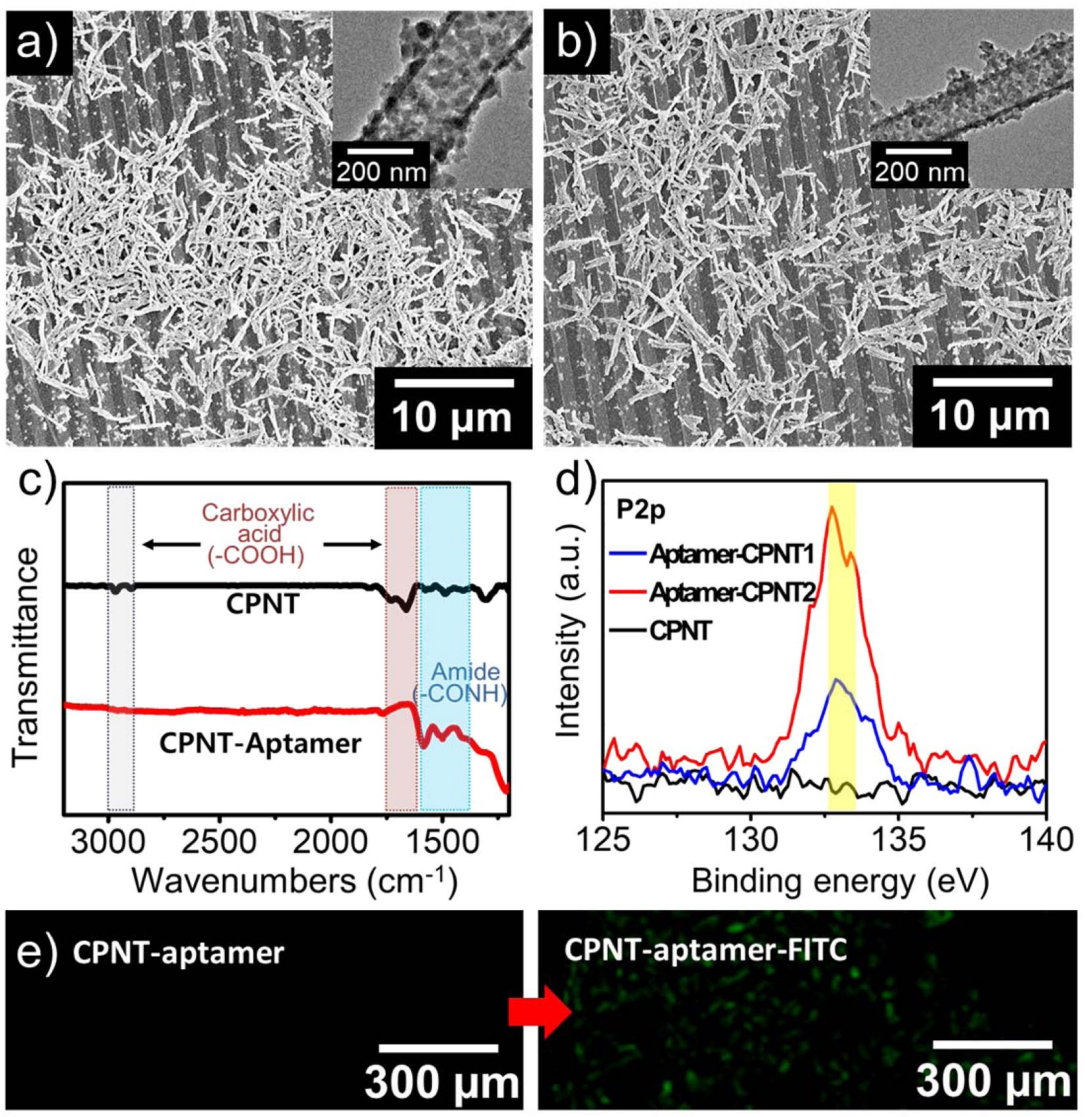
SEM images of CPNTs on IME arrays: (**a**) CPNT1 (*ca*. 200 nm in diameter) and (**b**) CPNT2 (*ca*. 120 nm in diameter). The insets show transmission electron microscopy (TEM) images of CPNT1 and CPNT2. (**c**) FT-IR spectroscopy of CPNTs and aptamer-CPNTs. (**d**) XPS analysis of P2p (*ca*.133 eV) of the aptamer-conjugated CPNT1 and CPNT2. (**e**) Fluorescent images of aptamer-CPNTs (right) and FITC-aptamer-CPNTs (left). The image of FITC was observed along the IME arrays.

To confirm the formation of the aptasensors, successful production of CPNTs *ca*. 200 nm in diameter (CPNT1) and *ca*. 120 nm in diameter (CPNT2) on the IME array was confirmed by the SEM images in Fig. 4a,b. While the reactivity of the Py-COOH monomer was significantly low for oxidative polymerization due to the presence of functional groups such as COOH, the addition of Py co-monomer accelerated the electrochemical polymerization^51^. Once the initiation step was triggered by iron chloride salt, the following reaction could be spontaneous and fast. The transmission electron microscopy (TEM) images in Fig. 4 clearly show that the morphology of the produced CPNTs is tubular and that each diameter is different (insets in Fig. 4a,b). It was obvious that a sufficient amount of CPNTs with different diameters was obtained by simple polymerization. This was important because an ample number of sites existed for the specific interactions between nanotubes (NTs), condensing agent, and aptamer molecules. It was predictable that the performance of the sensor was strongly dependent on the uniformity and dimension of the CPNTs. As the shape of the CPNTs was dominantly one-directional, the spacing between these NTs on the substrate was distant. Therefore, we supplied a sufficient amount of NTs to the electrode to guarantee intimate contact between NTs and the electrode. This was advantageous because the capacity of the CPNTs enabled to determine the sensitivity and selectivity of aptasensor.

To confirm the integration of DA aptamer on the CPNTs covalently, Fourier-transform infrared spectroscopy (FT-IR) and fluorescence image were introduced. First, the chemical characterization of aptamer-CPNTs was carried out using FT-IR(Alpha-p) spectroscopy (Fig. 4c). The absorption peaks of the CPNTs were characterized with the absorption peaks in the range of 1725 cm^−1^ to 1663 cm^−1^ which are contributed to stretching vibrations of acid group (RCOOH) on CPNT. The broad OH stretch of carboxylic acids on CPNT is also attributed around 3000 cm^−1^. However, the amide groups, which are formed by covalent bonding between CPNT and aptamer, enabled to be characterized by amide I (at 1620 cm^−1^) and II (at 1500 cm^−1^) bonds^52,53^. Generally, aptamer is composed of DNA which has a phosphate backbone. Therefore, X-ray photoelectron spectroscopy (XPS) analysis was introduced to compare the P2p ratios (*ca*. 133 eV) in aptamer-conjugated CPNT1 and CPNT2 (Fig. 4d). The P2p ratios of aptamer-CPNT2 is higher than one of the aptamer-CPNT1, resulted into the presence of more amount of the aptamer on the CPNT2^54^. Furthermore, the aptamer-CPNTs on IME were characterized by fluorescent images with following samples: aptamer-CPNTs and fluorescein isothiocyanate (FITC)-conjugated aptamer-CPNTs. The aptamer-CPNTs showed no significant fluorescent images (**left in** Fig. 4e), while the aptamer-CPNTs conjugated with FITC were clearly observed by fluorescence microscope (EVOS M5000) with emission spectrum peak wavelengths of approximately 519 nm (**right in** Fig. 4e).

**Figure 5:**
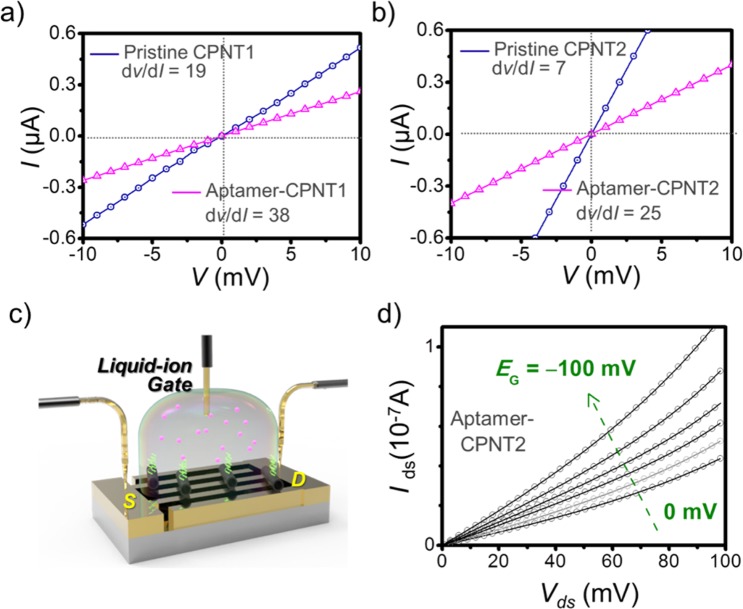
Current-voltage (*I*-*V*) curves of (**a**) CPNT1 and (**b**) CPNT2 before and after the introduction of aptamer. Characterization of liquid-ion gated aptasensors: (**c**) schematic illustration of liquid-ion gated aptasensors using CPNTs on the IME array. (**d**) Output characteristics of liquid-ion gated aptasensors with CPNTs.

The first step to characterizing the electrical properties of aptasensors was examining the electrical contact between sensing material aptamer-conjugated CPNTs and IME arrays. Figure 5a,b displays *I-V* curves of the CPNTs on the arrays obtained before and after the introduction of aptamer under ambient conditions. The slope variation (d*V*/d*I*) for CPNT2 (from 7 to 25) is larger than that for CPNT1 (from 19 to 38), which is caused by the different amounts of aptamer attachment on the CPNTs. A linear correlation over a voltage range of −10 to +10 mV (scan rate: 0.1 mVs^−1^) showed a stable ohmic contact. A slight decrease in the value of slope was attributed the increase in resistance due to the introduction of aptamer. It was also clear that the aptasensor could maintain reliable electrical contact to provide abundant conductive pathways. To measure electrical responses to DA, a liquid-ion gated aptasensor was constructed. The schematic illustration in Fig. 5c indicates that the sensing medium, aptamer-CPNTs, was surrounded with phosphate-buffered saline (PBS, pH 7.4) as the electrolyte. Preventing the leakage of liquid gate was critical for biosensor performance, which was overcome by adequate sealing with grease. This strategy was advantageous because it achieved both subtle contact with the CPNTs and signal amplification^55^.

Figure 5d presents the output characteristics of the liquid-ion gated FET aptasensor with CPNT2 operated under ambient conditions with *E*_G_ varying from 0 to −100 mV in −20 mV steps (source-drain voltage sweep rate = 1 mV^−1^). The drain to source current (*I*_*DS*_) increased with negatively increasing gate potential, indicating charge transport behavior. This observation was important because the binding of target molecules to the aptamer could be detected according to a change in the drain to source current under this p-type configuration. It could also be inferred that the aptamer-CPNTs were suitable as sensing devices for DA detection.

### Aptasensor performance

**Figure 6:**
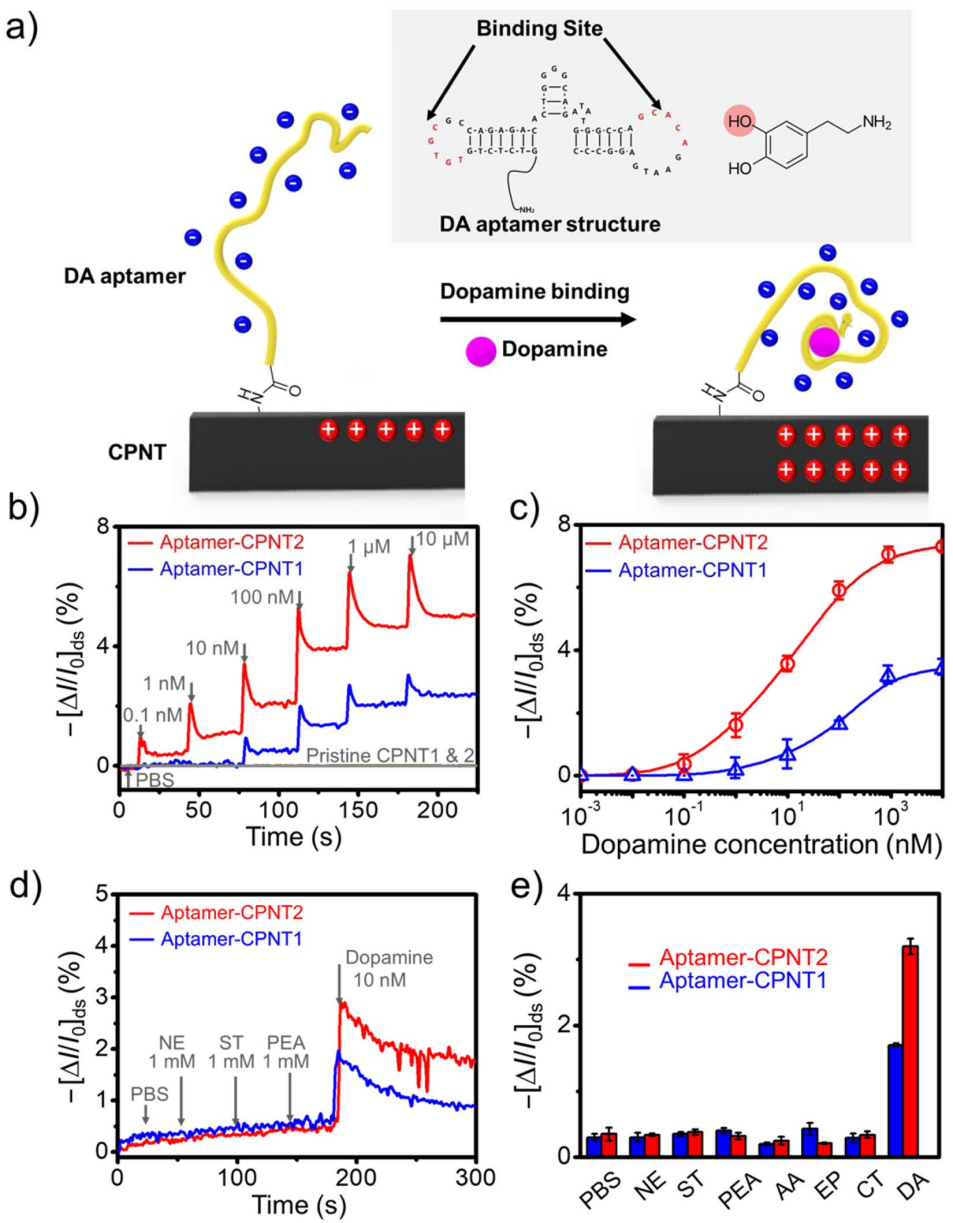
(**a**) Schematic illustration of the sensing mechanism between DA and DA aptamer (the inset shows the aptamer sequence with binding site and the red circle with a hydroxyl group in the position 3 of DA). (**b**) Real-time responses of liquid-ion gated aptasensors to various DA concentrations. (**c**) DA concentration-dependent real-time responses of aptasensors. (**d**) DA discrimination by aptasensors under NE, ST and PEA. (**e**) Extended selectivity of aptasensors toward various neurotransmitters: the most sensitive responses with DA. The error bars, which are in Fig. 6b,d, were calculated as standard deviation (n = 5).

As the successful fabrication of FET-type aptasensors was confirmed, the sensor performance, such as sensitivity and selectivity of the detection of real DA samples obtained from cells, was measured. First of all, although the crystal structure of the DA-binding aptamer is still unresolved, the sensing mechanism can be suggested with previous biochemical data; the major binding site between DA and aptamer is two major stem-loop domains (Fig. 6a)^50^. The aptamer benchmarked has a pairing interaction between the two major stem-loop domain, which are consisted of five nucleotides (C-G-T-G-T and G-C-A-C-A), to form the DA binding pocket. The binding pocket is supposed to interact with the hydroxyl groups of the position 3 in DA structure by hydrogen bonding^48–50^. The binding cites enable contact to be highly compact and enclosed (Fig. 6a **inset**)^56^. Therefore, the sensing mechanisms can be explained with two suggestions: 1) the DA molecules closely interact with two major stem-loop domains of negatively charged aptamers, and then, the DA-aptamer complex approaches the CPNT surface by physical or environmental movements. The close access of the negatively charged aptamer to the carrier, a hole, of CPNTs increases, resulting in an indirect doping effect. 2) The amount of complex increases with the negative charge intensity on the surface of CPNTs at pH 7.2 because the pK_a_ of DA is 6.2, increasing the negative charge intensity from the catechol side of DA. Figure 6b shows real-time responses from CPNT aptasensors toward various DA concentrations. First, pristine CPNT1 and 2 were introduced as control experiments and showed no significant responses. The sensitivity values of the CPNT2 aptasensor were higher than those of the CPNT1 aptasensor owing to the former’s enhanced surface area for the introduction of aptamers. The MDL of the CPNT2 aptasensor was *ca*. 100 pM, which is 100 times higher than that of the CPNT1 aptasensor. The CPNT aptasensor enables discrimination between healthy individuals and patients with DA-related illnesses, such as Parkinson’s disease and schizophrenia, because the patients have been determined to have DA concentrations of *ca*. 0.6 to 6.2 ng/mL (3.9 to 40.5 nM) by HPLC.

To quantify the sensing behaviors of CPNT aptasensors, the normalized sensitivity to various DA concentrations were evaluated, and dose-dependent DA concentrations were obtained, as shown Fig. 6c. The dose-dependent values indicate that the CPNT2 aptasensor has higher sensitivity changes than one of the CPNT1 aptasensor at a fixed concentration. In addition, the CPNT aptasensors were characterized by excellent sensitivity in the presence of similar neurotransmitters, such as norepinephrine (NE), serotonin (ST), and phenethylamine (PEA), and even at a high concentration of DA (10 mM) (Fig. 6d). The response from the CPNT2 aptasensor was also monitored at a higher sensitivity level than the CPNT1 aptasensor. Moreover, the extended selectivity of aptasensors was also measured using various neurotransmitters, including NE, ST, PEA, ascorbic acid (AA), catechol (CT), epinephrine (EP) and DA (Fig. 6e). There were no significant responses without DA, and the graph showing sensitivity levels clearly indicates that the aptasensors enable the discrimination of DA in mixtures.

**Figure 7:**
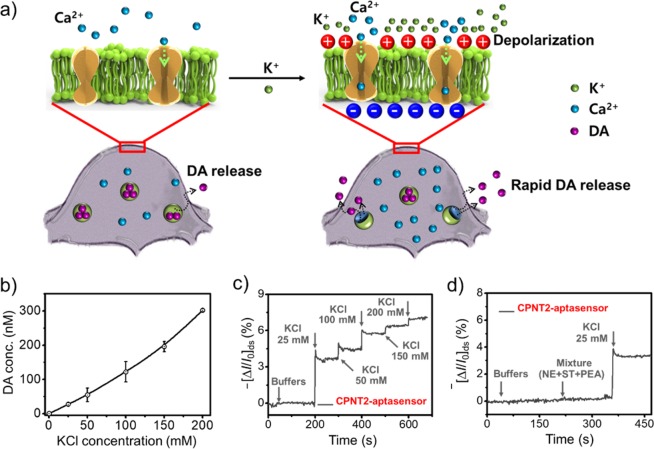
(**a**) Schematic illustration of DA release by Ca^2+^ and K^+^. The Ca^2+^ ion channels with K^+^ are more active than one without K^+^. (**b**) KCl concentration-dependent DA concentration produced by cell exocytosis (the error bars were calculated as standard deviation, n = 5). (**c**) Real-time responses of CPNT2-aptasensor to exogeneous DA samples released at various KCl concentrations. (**d**) Selectivity of CPNT2-aptasensor to exogeneous DA sample in mixtures.

To confirm exogeneous DA release from the PC12 cells, target samples with DA release were prepared by a DA release assay (see the experimental section for details). Various KCl concentrations (12.5 mM to 200 mM) were sequentially introduced to promote Ca^2+^ influx owing to membrane depolarization induced by K^+^ charge, leading to the rapid DA release from PC12 cell (Fig. 7a). Therefore, the amount of the active Ca^2+^ ion channel in the cell with K^+^ is larger than one from the cell without K^+^. The concentrations of DA in target and control samples were calculated by commercial DA ELISA (Enzyme-Linked Immunosorbent Assay) kits (KA 1887, Abnova), resulted into the increasing DA concentrations dependent on the KCl concentrations (Fig. 7b). Based on the results with DA exocytosis, the increasing KCl concentration induced rapid and high-level DA release under a constant KCl treatment time. To investigate DA exocytosis with the sensitivity and selectivity of CPNT aptasensors, the prepared exogeneous DA samples were characterized. Figure 7c displays KCl concentration-dependent real-time DA responses with the addition of various exogeneous DA samples. The response levels from CPNT2 aptasensor increased with increasing KCl concentration, showing similar standard DA concentration-dependent results. Moreover, the CPNT2 aptasensor clearly displayed excellent selectivity toward DA in DA release samples, even in mixtures (Fig. 7d).

## Conclusion

In this study, a DA exocytosis system using CPNTs was firstly demonstrated by a liquid-ion gated FET system. The aptasensors were prepared by conjugating CPNTs with designed diameters (*ca*. 120 nm and *ca*. 200 nm) and DA-selective aptamers. Introducing a liquid-ion gated FET system resulted in high-performance DA aptasensors. The aptamer on the CPNTs was operated as a gate-modulator toward DA in this system. Therefore, the gate-modulated stimulation from DA was monitored and showed highly sensitive (*ca*. 100 pM) and selective DA detection. Moreover, the CPNT aptasensors also demonstrated excellent analysis of DA exocytosis by detecting exogeneous DA samples prepared by a DA release assay. Based on the excellent sensing properties of FET-type aptasensors, the aptasensors can be used for clinical diagnostic studies by overcoming i) DA extraction from the patient samples such as blood and saliva without additional pre-treatments and ii) cost-efficiency.

## Materials and Methods

### Materials

Pyrrole (Py, 98%) and pyrrole-3-carboxylic acid (Py-COOH, 95%) were purchased from the Aldrich and Acros Organics. A surfactant, sodium bis(2-ethylhexyl)-sulfosuccinate (AOT, 98%) and hexane (98%) were also purchased from the Aldrich. The conjugation reagent, 4-(4,6-Dimethoxy-1,3,5-triazin-2-yl)−4-methyl-morpholinium chloride (DMT-MM, 96%) was purchased from Aldrich. The aptamer was purchased from Bioneer. Inc.

### Fabrication of carboxylated polypyrrole nanotubes (CPNTs)

CPNTs with different diameters (*ca*. 120 and 200 nm) were prepared by benchmarks in our previous works^51^. A surfactant, AOT (15 mmol), was dissolved in hexane (40 mL), and the solution was stirred for 30 minutes to reach equilibrium. Subsequently, aqueous FeCl_3_ solution (7 M, 1 mL) was introduced into the AOT/hexane solution to generate reverse-cylindrical micelles containing iron cations^55^. Finally, a pyrrole (Py) (3.75 and 7.5 mmol) and carboxylated pyrrole (Py-COOH) (0.25 mmol) mixture was added dropwise into the reverse-cylindrical micelle phase. The chemical oxidation polymerization of Py/Py-COOH mixture monomers proceeded for 3 h at 18 °C to room temperature^51^. The resulting product was thoroughly washed with excess ethanol to remove the surfactant and other residual reagents. The final products were obtained after drying under vacuum at room temperature.

### Instruments and procedures for IME array preparation

The photoregistor (DNR L300) was coated by a spincoater (ACE-200) on a glass wafer. Aligner (MA-6 II) was used to develop the photoregistor. To design the electrodes, Au/Cr was deposited on the photoregistor-coated glass wafer with a thickness 25/5 nm by Thermal Evaporator (MHS-1800). The Au/Cr deposited on glass wafer was developed into the developer, resulted into the IME array electrodes. Finally the glass wafer was cutted into each electrode substrates by Dicing Saw (DAD 3350). All processes were conducted by ourselves at the Inter-university Semiconductor Research Center (ISRC).

### Construction of FET-type aptasensors

The substrate was treated with a 1 wt% aq. amino silane (3-aminopropyltrimethoxysilane, APS from Aldrich) solution for 12 h to introduce amino groups on the IME glass substrate and washed with distilled water. The amino group-functionalized IME substrate was exposed to a mixture solution containing 3 wt% CPNT solution (40 µL) and 1 wt% aq. DMT-MM solution (40 µL) for 12 h. The IME array substrate conjugated with CPNTs was washed with distilled water several times. Subsequently, the aptamer was then immobilized onto the CPNT surface on IME substrate with same reaction: the mixture of aptamer and DMT-MM solution (40 µL) was dropped on the CPNT-IME substrate for 12 h. Afterwards, the final aptasensor was rinsed with distilled water and dried at room temperature under glass container. The characterization of the aptasensor was carried out using FT-IR (Alpha-p), TEM (MTE20), SEM (MSE20), SEM-EDX (MSE20), XPS (PHI 5000 VersaProbe (Ulvac-PHI)), fluorescence image (EVOS M5000) and *I*-*V* curve (Keithley 2612 A).

Reaction 1

$$\begin{array}{c}{\rm{Hydrolysis}}:{H}_{2}N{(C{H}_{2})}_{3}Si{(OC{H}_{3})}_{3}+3{H}_{2}O\to {H}_{2}N{(C{H}_{2})}_{3}Si{(OH)}_{3}+3C{H}_{3}OH\\ {\rm{Condensation}}:{H}_{2}N{(C{H}_{2})}_{3}Si{(OH)}_{3}+3OH-{\rm{substrate}}\to {H}_{2}N{(C{H}_{2})}_{3}Si{(O)}_{3}-{\rm{substrate}}\end{array}$$

Reaction 2

$${\rm{Condensation}}:Py-COOH+{H}_{2}N{(C{H}_{2})}_{3}Si{(O)}_{3}-{\rm{substrate}}\to Py-CONH{(C{H}_{2})}_{3}Si{(O)}_{3}-{\rm{substrate}}$$

### Dopamine detection experiments

The aptasensor was placed on the stage of the probe station connected Keithley 2612 A. *I-V* curves were measured by the voltage scan rate (0.1 mVs^−1^). To make liquid-ion gate, the glass chamber was covered on the aptasensors and phosphate-buffered saline (PBS, pH 7.4) was fulfilled into it. The liquid-ion gated aptasensor was characterized as FET by operating source and gate voltages. The output characteristics of the FET-aptasensors were carried out under ambient conditions with *E*_*G*_ varying from 0 to − 100 mV in −20 mV steps (*V*_*DS*_ sweep rate = 1 mV^−1^). Real-time responses were monitored by sequentially dropping DA samples (*ca*. 2 µL) with standard DA samples (from 0.1 nM to 10 µM) and exogeneous DA samples (from KCl 25 mM to 200 mM) under fixed source and gate voltages (*V*_*DS*_ = 50 mV and *E*_*G*_ = −50 mV).

### DA release assay

Rat pheochromocytoma PC12 cell lines were obtained from the American Type Culture Collection (ATCC). PC12 cells were cultured in RPMI-1640 in 100 Φ dishes using 10% horse serum (v/v) (heat-inactivated, Gibco), 5% fetal bovine serum (v/v) (heat-inactivated, Gibco) and 1% penicillin-streptomycin (Gibco) solution. Then, the cells were separated by incubation at 37 °C for 2 minutes using 1 mL of Trypsin-EDTA 0.25% solution (Gibco). The detached cells were transferred to 24-well plates at 5 ×10^4^ in each well and incubated at 37 °C.

The DA was pre-equilibrated in PC12 cells: the cells were confirmed to be well adhered by microscope, and the medium was removed. One milliliter of loading buffer (Hank’s Balanced Salt Solution with 10 mM HEPES) was added to each well and incubated at 37 °C for 2 minutes. The supernatant was discarded, and then DA hydrochloride (20 nM) was prepared in loading buffer. One milliliter of DA hydrochloride solution was added to each well. The samples were incubated for 20 minutes at 37 °C, and the DA was pre-equilibrated with PC12 cells. The samples were washed with loading buffer (0.5 mL) 3 times.

KCl stimulation and supernatant acquisition: KCl buffer was prepared at 5 concentrations (25 mM, 50 mM, 100 mM, 150 mM and 200 mM) in loading buffer. PC12 cells were exposed to 1 mL of KCl buffer (25 mM, 50 mM, 100 mM, 150 mM and 200 mM) in each well and incubated for 20 minutes at 37 °C. After K^+^ stimulation, PC12 cell supernatant was collected into an appropriately labeled 1.5 mL tube.

### Dopamine detection using ELISA kits

The DA concentrations from exogeneous samples were calculated by commercial DA ELISA (Enzyme-Linked Immunosorbent Assay) kits (KA1887, Abnova) with manual. There are two steps with ELISA kits: i) extraction and acylation and ii) DA ELISA (see the specific process information at http://www.abnova.com/products/products_detail.asp?catalog_id=KA1887). The exogeneous samples were treated by the commercial kits with the two steps, resulted into increasing DA concentrations depending on the KCl concentrations.
